# The Effect of Web-Based Telerehabilitation Programs on Children and Adolescents With Brain Injury: Systematic Review and Meta-Analysis

**DOI:** 10.2196/46957

**Published:** 2023-12-25

**Authors:** Zeyu Wang, Kang He, Xin Sui, Jiang Yi, Zhaoyun Yang, Kai Wang, Yan Gao, Linfang Bian, Junjie Jiang, Lijing Zhao

**Affiliations:** 1 School of Nursing Jilin University Changchun China; 2 The Second Hospital of Jilin University Jilin University Changchun City China

**Keywords:** acquired brain injury, web-based, telerehabilitation, motor function, physical activity level, lower limb strength, children, adolescents, meta-analysis

## Abstract

**Background:**

Acquired brain injury (ABI) in children and adolescents can lead to motor and executive impairments that often require long-term treatment. The implementation of web-based telerehabilitation therapy at home is a method to improve the functional status of patients. Therefore, we performed a systematic review of the effects of web-based telerehabilitation programs on functional outcomes in children and adolescents with brain injury and supplemented the findings with a meta-analysis.

**Objective:**

This study evaluated the therapeutic effect of web-based telerehabilitation training on children and adolescents with brain injury to determine whether web-based telerehabilitation therapy improved motor function, executive function, physical activity level, lower limb strength, hand and upper limb function, visual processing skills, and occupational functional performance in children and adolescents with brain injury.

**Methods:**

PubMed, Embase, Scopus, Web of Science, and the Cochrane Library were searched for randomized controlled trials on web-based telerehabilitation programs in children and adolescents with brain injury until December 2022, and the risk of bias was evaluated using the Cochrane Collaboration Tool. Relevant data were extracted, and a meta-analysis was performed using RevMan5.3 software.

**Results:**

Overall, 17 studies involving 848 patients were included. Web-based telerehabilitation therapy improved the motor function (standardized mean difference [SMD] 0.29, 95% CI 0.01-0.57; *P*=.04), physical activity level (SMD 0.42, 95% CI 0.11-0.73; *P*=.007), lower limb strength (SMD 0.52, 95% CI 0.13-0.90; *P*=.009), and visual processing skills (SMD 0.26, 95% CI 0.02-0.50; *P*=.04) of children and adolescents with brain injury. It also improved executive function in letter-number sequencing (SMD 1.26, 95% CI 0.26-2.26; *P*=.01), attention (SMD 0.38, 95% CI 0.09-0.66; *P*=.009), and symbol search (SMD 1.18, 95% CI 0.43-1.93, *P*=.002).

**Conclusions:**

Web-based telerehabilitation therapy improved motor function, physical activity level, lower limb strength, letter-number sequencing, attention, and symbol search, which improved the quality of life in children and adolescents with brain injury. Web-based telerehabilitation programs provide great convenience for children and adolescents with ABI who need long-term treatment and allow them to exercise at home for rehabilitation training. The widespread implementation of remote interventions also provides children and adolescents in remote areas with better access to rehabilitation services. This review provides evidence for the effectiveness of web-based telerehabilitation therapy, but there was heterogeneity in some of the results because of different disease types and intervention programs. Future studies can expand the sample size according to disease type and increase follow-up time according to different exercise prescriptions to further refine the long-term effects of this intervention on various functions of children and adolescents with ABI.

**Trial Registration:**

PROSPERO CRD42023421917; https://www.crd.york.ac.uk/prospero/display_record.php?RecordID=421917

## Introduction

### Background

Acquired brain injury (ABI) is a major cause of morbidity and mortality worldwide. ABI can be caused by traumatic injuries (traffic accidents, accidental falls, sports injuries, and violence) or ischemic brain injuries (ischemic stroke, cerebral ischemia secondary to cardiac arrest, neurological damage after cardiopulmonary bypass, and perinatal ischemic hypoxic encephalopathy) [1,2]. ABI in children and adolescents is defined as a brain insult acquired after the first year of life [2]. ABI includes traumatic brain injury and nontraumatic brain injury, with incidence rates of 274 to 317 per 100,000 [3-5] and 82.3 per 100,000 [6], respectively.

Most patients with ABI have a wide range of neurological sequelae, such as motor deficits, cognitive dysfunction, hand and upper limb dysfunction, language and speech abnormalities, and behavioral problems [7]. Hand dysfunction includes motor skill, speed, and coordination disorders [8]. Depending on the severity of trauma, some children and adolescents may have persistent deficits in visual processing skills [7], and these disorders have a lifelong dynamic impact on health and well-being [9]. Therefore, we should focus on these disorders in children and adolescents with ABI. However, treatment of these disorders often requires long-term care and follow-up, which is difficult. Therefore, this review aimed to assess the effectiveness of web-based telerehabilitation programs in children and adolescents with brain injury.

With the rapid development of internet technology, the World Health Organization has recognized the potential of mobile technology to change the nature of medical services [10]. Telerehabilitation fills the defects of insufficient opportunities for follow-up because of long distances or high costs [11,12], and patients can assess their own barriers and develop corresponding training plans by conducting rehabilitation programs at home [13]. These advantages provide an opportunity to facilitate the treatment of children and adolescents with ABI, which keeps these individuals safe, improves outreach, and ensures compliance with rehabilitation programs [13,14].

A review by Corti et al [15] described interventions for tele-technology training for children and adolescents with ABI and found significant effects after telerehabilitation interventions targeting cognitive and behavior disorders, suggesting that telerehabilitation at home is feasible [16,17]. However, some of the current telerehabilitation interventions are limited by expensive equipment [18] or lack of supervision, which leads to low patient participation in rehabilitation or unsatisfactory intervention effects [15]. Linden et al [19] performed a meta-analysis of technology-based training programs for children and adolescents with ABI. Only one report showed significant differences between the intervention and control groups in improving problem-solving and sentence recall, which are important components of executive function. However, these findings may be limited by the small number of participants included in the study, insufficient information, and limited measures of outcome assessments. Wade et al [20] reported that a technology-assisted intervention effectively improved behavioral problems and executive function in children and adolescents with brain injury. However, these results were limited by the small sample size, heterogeneity, and lack of a control group. A review by Beckers et al [21] reported on the efficacy of home regimens for children and adolescents with cerebral palsy of the upper limbs and showed that family therapy programs for children and adolescents with cerebral palsy were feasible, but a meta-analysis could not be performed because of expected clinical and methodological heterogeneity.

In summary, several articles were published on telerehabilitation interventions for children and adolescents with ABI, but the effectiveness of internet-based teletherapy at home has not been clearly defined because of small sample sizes, lack of control groups, and the heterogeneity of research methods.

### Aims of This Review

This study reviewed randomized controlled studies of web-based teletherapy interventions and analyzed the data of various outcomes after web-based intervention in children and adolescents with ABI, including motor function, executive function, physical activity level, lower limb strength, hand and upper limb function, visual processing skills, and occupational function performance, to provide evidence-based rehabilitation options that use the internet for new treatment strategies for children and adolescents with brain injuries.

## Methods

### Study Registration

This systematic review and meta-analysis is reported in accordance with the PRISMA (Preferred Reporting Items for Systematic Reviews and Meta-Analyses) statement [22]. An initial review protocol was preregistered on PROSPERO (CRD42023421917).

### Literature Search Strategies

Computer retrieval was the main method used, and the search databases included PubMed, Embase, Cochrane Library, Web of Science, and Scopus. The period considered for inclusion in the study ranged from the establishment of each database to December 2022. There was no limit to the type of literature, which included journal literature, conference papers, and dissertations.

Searches were performed using a combination of Medical Subject Headings terms and entry terms. The corresponding retrieval formula is formulated according to the characteristics of each database (Textbox 1).

**PubMed retrieval.**

**Consider PubMed as an example**

- (Telerehabilitation[MeSH Terms]) OR (Telerehabilitation[Title/Abstract])) OR (Tele-rehabilitation[Title/Abstract])) OR (Tele rehabilitation[Title/Abstract])) OR (Tele-rehabilitations[Title/Abstract])) OR (Remote Rehabilitation[Title/Abstract])) OR (Rehabilitation, Remote[Title/Abstract])) OR (Rehabilitations, Remote[Title/Abstract])) OR (Remote Rehabilitations[Title/Abstract])) OR (Virtual Rehabilitation[Title/Abstract])) OR (Rehabilitation, Virtual[Title/Abstract])] OR [Rehabilitations, Virtual[Title/Abstract]]) OR (Virtual Rehabilitations[Title/Abstract])) OR (web)) OR (home)) AND ((((((((brain) OR (cranial)) OR (cerebral)) OR (head)) OR (intracranial)) OR (fossa)) OR (subarachnoid)) OR (cerebrocranial))) AND (((children) OR (pediatric)) OR (adolescents)).

Multimedia Appendix 1 shows specific search formulas.

### Study Selection Process

Two review authors independently selected studies according to the inclusion and exclusion criteria and discussed or consulted third-party opinions when there was disagreement. Duplicate studies were first removed from literature screening, and titles and abstracts were read to exclude irrelevant studies. After removing articles that were not randomized controlled trials (RCTs), the full text was read to obtain the final included studies.

### Literature Inclusion Criteria

The following criteria were used for inclusion in the review:

1. Diagnosis of ABI (included in the category of acquired brain damage of a traumatic nature, eg, falls, assaults, sports injuries, pedestrian injuries, and bicycle or motorcycle crashes) and nontraumatic injuries (eg, stroke, infectious disease, brain tumors, lack of oxygen, and toxic exposure). Patients with acquired brain damage were considered eligible for the review only if they were in a chronic phase (ie, at least 1 year after injury).
2. Children and adolescents (aged <18 years).
3. Able to follow instructions sufficient to participate in simple computer games.
4. Able to access the internet at home (ie, phone line or internet access).
5. RCTs.
6. The included literature evaluated the effectiveness of web-based telerehabilitation programs for children and adolescents with brain injury (including web-based interventions provided training equipment for patients to use at home) by comparing with no training or usual care. Rehabilitation programs may include telemedicine rehabilitation training, virtual game training at home, computer-assisted training modules, and robotic devices that may be used at home under third-party guidance or independently [19].
7. All participants were divided into 2 groups: the intervention group (web-based telerehabilitation) and the control group (no training or usual care).

### Exclusion Criteria

The exclusion criteria for the study subjects were as follows: (1) progressive neurological disorder, severe concurrent illness, or disease not typically associated with ABI; (2) severe sensory, motor, cognitive, and visual deficits that could not be corrected using compensatory tools and interfered with training execution and assessment; (3) a diagnosis of photosensitive epilepsy, as computer-based stimulation could produce negative health effects in these patients; and (4) any surgical or medical intervention in the 6 months before starting the study.

Literature exclusion criteria were as follows: (1) not English literature, (2) not using internet intervention at home, (3) duplicate published literature, (4) unavailable full text or unable to extract data, and (5) animal experiments.

### Outcome Measures

The primary outcome measure in the literature was motor function. The evaluation tools included the Gross Motor Function Measure-66 (GMFM-66), Bruininks-Oseretsky Test of Motor Proficiency (BOTMP), and Assessment of Motor and Process Skills (AMPS).

The secondary outcomes included physical activity level, lower limb strength, visual processing skills, executive function, hand and upper limb function, balance function, and the Canadian Occupational Performance Measure (COPM).

### Data Extraction

Two independent review authors extracted and checked the data, which included basic information on the included studies (first author, year, and country), data profile (sample size, mean, and SD), interventions (type and duration of follow-up), and outcome measures.

### Literature Quality Assessment

The methodological quality of the included studies was assessed using the Cochrane Collaboration Tool [23,24]. The scale items were divided into 7 areas: random sequence generation (selection bias), allocation concealment (selection bias), blinding of participants and personnel (performance bias), binding of outcome assessment (detection bias), incomplete outcome data (attrition bias), selective reporting (reporting bias), and other biases. Each criterion was rated as low, high, or unclear risk [25]. To draw conclusions about the overall risk of bias within or across trials, we summarized assessments across items in the tool for each outcome within each trial [24]. Two independent investigators performed the assessments and discussed or consulted third parties when the results were inconsistent.

### Certainty Assessment

The quality of evidence was evaluated using the Grading of Recommendations Assessment, Development, and Evaluation (GRADEpro GDT, web-based version) professional guideline development tool. RCTs start with a “high” quality of evidence, and the quality of evidence is upgraded or downgraded based on the following assessment: (1) risk of bias, (2) inconsistency, (3) indirectness, (4) imprecision, and (5) publication bias [26].

### Publication Bias

Publication bias was assessed visually using a funnel plot and statistically using the Egger test [27].

### Statistical Analysis

RevMan5 software [28] was used for statistical analyses. For studies included in the meta-analysis, the sample size, mean, and SD of the primary outcomes were extracted for the intervention and control groups. When the measurement method or unit was inconsistent, a standardized mean difference was used. Because the outcome assessment scales were different, a random-effects model was used for data analysis. The Cochrane Q (chi-square test) and *I*^2^ statistics were used to evaluate the heterogeneity of the included studies. The significance level for the chi-square test was set at *P*<.10, indicating heterogeneity. An *I*^2^ value of 0% to 40% was considered not important, 30% to 60% indicated moderate heterogeneity, 50% to 90% indicated substantial heterogeneity, and 75% to 100% indicated considerable heterogeneity [29].

### Subgroup Analysis

Subgroup analyses were performed to test for differences in scoring scales (GMFM-66, AMPS, and BOTMP), intervention duration (<12 and ≥12 weeks), interaction with medical professionals (interaction and no interaction), test sample size (<50 and ≥50 participants), and nation (Asian, Europe, Oceania). According to the Cochrane Handbook [30], the typical advice for undertaking simple regression analyses is that at least 10 observations (ie, 10 studies in a meta-analysis) should be available for each characteristic modeled.

### Sensitivity Analysis

In the case of considerable heterogeneity, a sensitivity analysis was performed, a Galbraith plot was constructed, outliers were identified by omitting one study at a time from the study pool, and their corresponding level of heterogeneity was rechecked.

## Results

### Results of the Literature Search

The literature search identified a total of 52,022 records on December 3, 2022. A total of 9530 records were collected from PubMed, 9406 from Scopus, 21,066 from Embase, 1382 from the Cochrane Library and 10,638 from the Web of Science databases.

After removing duplicates (n=27,263), the titles of 23,867 records were screened. On the basis of the title, 23,499 records were excluded because of irrelevant content or formatting. A careful reading of the full text removed 351 articles, and 17 articles were finally included after 2 researchers independently reached a consensus.

**Figure 1:**
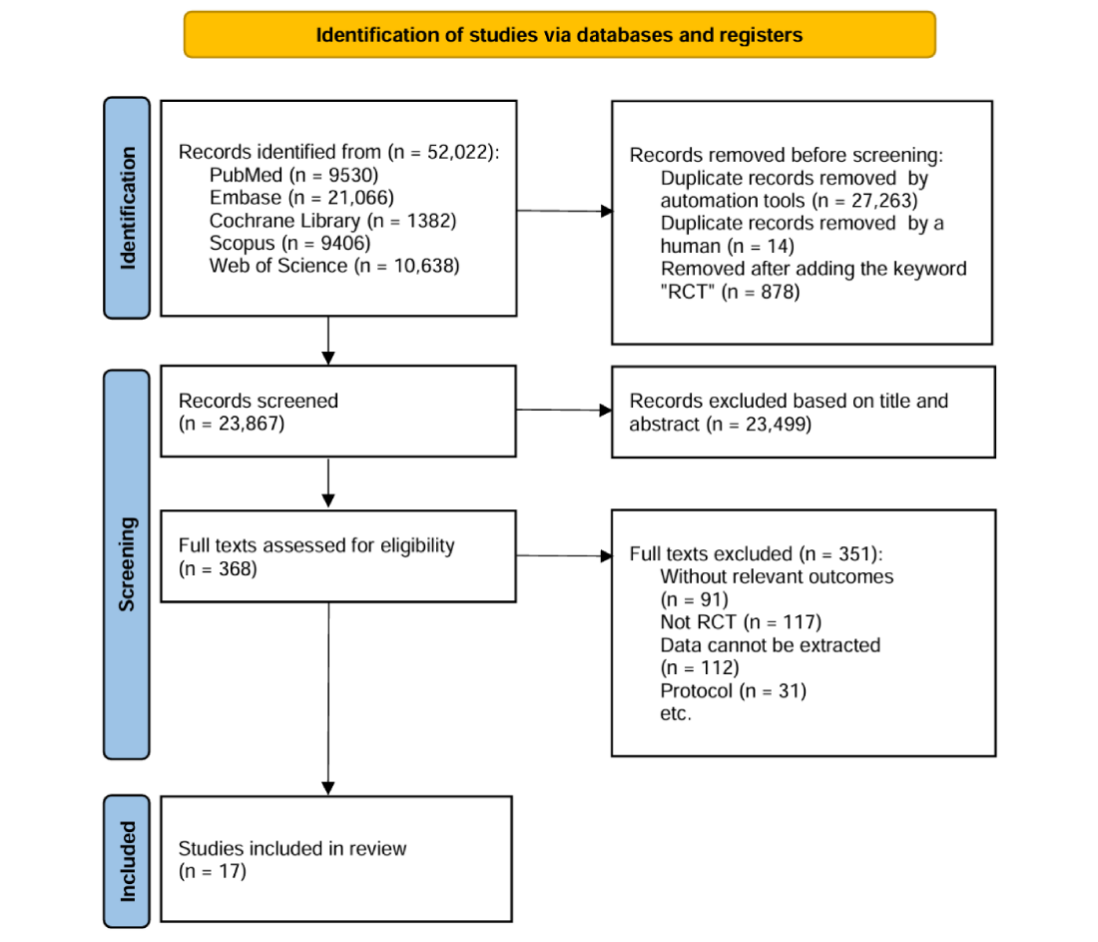
Flowchart for study identification, screening, eligibility, and inclusion.

The literature screening process and results are shown in Figure 1.

### Basic Characteristics of the Included Studies

The definitive analysis of this systematic review included 17 studies published between 2012 and 2022 that reported on web-based telerehabilitation interventions for 848 children and adolescents with brain injury from China, Australia, and 4 other countries. Table 1 shows the basic characteristics of the 17 included studies [31-47]. The categories of ABI included were diverse (eg, cerebral palsy, brain tumors, and other types of brain injuries). Except for one study that included children aged <5 years, participants in the other studies were distributed from ages 6 to 18 years.

**Table 1:** The basic characteristics of the included studies.

Study	Nation	Group		Age (years)	Disease	Inclusion criteria	Exclusion criteria
		Intervention, n	Waitlist control, n				
Chen et al [31]	China	13	15	6-12	CP^a^	Diagnosis with CP with GMFCS^b^ levels I–II Age 6-12 years In prepubertal stage, ability to walk independently, ability to undergo a motor function and isokinetic muscle test, and ability to comprehend commands and cooperate during an examination	Chromosomal abnormalities Progressive neurological disorder or severe concurrent illness or disease not typically associated with CP Active medical conditions such as pneumonia Any major surgery or nerve block in the preceding 3 months Hormonal disturbance; Poor tolerance of isokinetic test or a poor cooperation during assessment
Chiu et al [32]	China	32	30	6-13	CP	Diagnosed with CP before 5 years of age were spastic hemiplegic; Age 6-13 years; Enough hand function to hold the Wii remote control.	Severe cognitive or visual problems
Conklin et al [33]	America	34	34	8-16	ALL^c^ or BT^d^	BT or ALL survivors who received cranial irradiation or intrathecal chemotherapy and had completed treatment at least 1 year before, without disease recurrence English speakers and ages 8-16 years, with IQ 70	History of premorbid central nervous system injury or disease (eg, traumatic brain injury, epilepsy) Preexisting attention-deficit/hyperactivity disorder, psychotropic medications within 2 weeks of enrollment, motor or sensory deficit precluding valid testing or completion of the intervention Psychological condition precluding or taking precedence over cognitive intervention
Corti et al [34]	Italy	18	14	11-16	ABI^e^	Nonprogressive ABI (eg, traumatic brain injury, stroke, anoxia, meningitis, encephalitis, postsurgical meningioma, and acoustic neuroma) In chronic phase (at least 1 year after the event) Age 11-16 years Speak Italian as a primary language	A previous diagnosis of psychiatric or cognitive problems Severe visual, auditory, or motor deficits Undergoing a parallel cognitive rehabilitation treatment A diagnosis of photosensitive epilepsy
Farr et al [35]	England	15	15	5-16	CP	Diagnosis with CP with GMFCS levels I-II Age 5-16 years Able to walk independently over short distances without use of walking aids Able to follow simple task instructions	With epilepsy who were photosensitive or had a seizure within the previous year Take anticonvulsant medication
Baque et al [36]	Australia	29	29	8-16	ABI	At least 12 months post-ABI, which was acquired ≥28 days post–full-term birth Age 8-16 years GMFCS levels I-II Cooperative and had sufficient comprehension, attention and concentration, visual and verbal abilities	A degenerative or metabolic condition Unstable epilepsy Undergone upper or lower limb surgery or any medical intervention in the past 6 months
Lai et al [37]	America	23	26	10-19	CP	Diagnosis with CP The ability to exercise with arms Age 10-19 years Access to a Wi-Fi internet connection at home Ability to use a device capable of viewing internet video content	Physically active (defined as >60 min/d of moderate to vigorous intensity exercise in a typical week) Complete blindness or deafness
Sakzewski et al [38]	Australia	29	29	8-16	ABI	A minimum of 12 months after ABI with GMFCS levels I-II Age 8-16 years Sufficient cooperation, cognitive understanding, visual acuity, and verbal communication	Unstable brain injury Unstable epilepsy undergone active medical treatment in the past 6 months Undergone surgical intervention in the 6 months before study commencement No persistent physical impairment after ABI
Tianming [39]	China	20	20	1.5-4.8	CP	Diagnosis with CP Ability to help stand and walk Able to understand and match instructions given Maintain combination therapy for 12 weeks or more	CP with other inherited diseases, metabolic diseases, and severe cardiopulmonary diseases; Selective posterior rhizotomy, peripheral nerve constriction and tendon lengthening of both lower limbs; 6 months to take antispasmodic drugs or botulinum toxin injection Those who do not cooperate with the treatment
M Piovesana et al [40]	Australia	51	50	8-18	CP	GMFCS levels I-II Age 8-18 years Sufficient cooperation and cognitive understanding	Received upper limb or lower-limb surgery in the previous 6 months Unstable epilepsy A respiratory, cardiovascular, or other medical condition
Mitchell et al [41]	Australia	51	50	8-17	CP	Diagnosis with unilateral CP with GMFCS levels I-II Age 8-17 years	Unstable epilepsy or medical conditions Underwent upper limb botulinum neurotoxin A injections or surgery in the previous 2 months or 6 months respectively
Pin and Butler [42]	China	9	9	6-14	CP	Diagnosis with CP with GMFCS levels III-IV Age 6-14 years Able to follow instructions	Had epilepsy or seizures Were unable to sit for long enough or were regular users of balance board type interactive computer games at home
Piovesana et al [43]	Australia	29	29	8-16	ABI	Diagnosis with ABI with GMFCS levels I-II Age 8-16 years Sufficient cognitive understanding, visual and verbal abilities, and cooperation Able to access the internet at home	Unstable epilepsy A degenerative or metabolic condition Undergone any surgical or medical intervention in the 6 months before starting the study
Sabel et al [44]	Sweden	7	6	7-17	BT	Age 7-17 years Completed treatment including RT^f^ for a BT between 1 and 5 years earlier	Uncontrolled seizures, severe motor or visual impairment, or severe autism Received tumor treatment Unable to speak Swedish
Sabel et al [45]	Sweden	7	6	7-17	BT	Age 7-17 years Completed treatment including RT for a brain tumor between 1 and 5 years earlier	Not in clinical remission or stable disease Severe intellectual disability, severe autism, photosensitive seizures; Not Swedish-speaking “Other disease/condition” included severe autism
James et al [46]	Australia	51	50	8-18	CP	GMFCS levels I-II Age 8-18 years Sufficient cooperation and cognitive understanding Internet access at home	Received upper- or lower-limb surgery in the previous 6 months Unstable epilepsy Respiratory, cardiovascular, or other medical condition
Wang e al [47]	China	9	9	5-12	CP	Age 5-12 years No excessive muscle tone No severe perceptual disorders No injections of botulinum toxin type A or operations on the affected hand within 6 months	Severe perceptual disorders Injections of botulinum toxin type A or operations on the affected hand within 6 months

^a^CP: cerebral palsy.

^b^GMFCS: Gross Motor Function Classification System.

^c^ALL: acute lymphoblastic leukemia.

^d^BT: brain tumor.

^e^ABI: acquired brain injury.

^f^RT: radiation therapy.

The treatment protocols for web-based remote interventions included in this study were different. The main intervention methods, evaluation indicators, and intervention effects in each study are presented in Table 2. There were various intervention methods (ie, training in a variety of game programs, including Mitii and Wii Fit or home-based virtual bicycle training), and the intervention time and frequency were different. Specific exercise prescription parameters have not been clearly defined. According to the RCT articles included in this study, the main treatment prescription was computer game training, 30 minutes each time, 3 to 6 times per week for more than 8 weeks [31,33-36,38-41,43-47].

**Table 2:** The main intervention methods, evaluation indicators and intervention effects of the included studies were analyzed.

Included studies	Type of intervention	Therapeutic parameters	Projects for intervention	Time of assessment	Outcome measures	Effect of intervention
Chen et al [31]	Internet-based cycling training at home	40 min/day, 3 times per week for 12 weeks	A 5 min warm-up exercise, 20 repetitions of sitting-to-standing movements, cycling for 20 min, and a cool-down exercise for 5 min.	Week 0, week 12	Gross motor function of the BOTMP^a^, muscle strength	The knee muscle strength of children with cerebral palsy in the treatment group was enhanced.
Chiu et al [32]	Home-based Wii Sports Resort training plus usual therapy	40 min/day, 3 times a week for 6 weeks	Four Wii Sports Resort games, from easiest to hardest—bowling, air sports, frisbee, and basketball.	Week 0, week 6, and week 12	Coordination, strength, hand function, carers’ perception of hand function	The treatment group had higher grip strength than the control group.
Conklin et al [33]	Computerized cognitive training programs	25 training sessions at home, 30-45 min/session for 5-9 weeks	Visual-spatial and verbal WM^b^ exercises presented as games. Participants demonstrating slower-than-desired progress were offered 5 additional sessions.	Pre- and posttraining	Working memory, attention, processing speed, executive dysfunction	Working memory, attention, and processing speed improved in the treatment group and executive dysfunction was greatly reduced.
Corti et al [34]	Remote computerized cognitive training	20 min/day, 5 times a week in 8 weeks	5 games were chosen for this study, each stimulating one of the target cognitive domains. Each game was used twice a day for a total of 10 daily exercises.	Week 1, week 10, week 19, and 6 months	Visual-spatial working memory, cognitive flexibility, arithmetic calculation, problem-solving, psychological adjustment	Working memory and arithmetic speed improved in the treatment group.
Farr et al [35]	Wii Fit active computer games	30 min, 3 times per week for 12 weeks	Nintendo Wii Fit plus games (12 programs)	Week 0, week 6, and week 12	Gross Motor Function Measure-66, the TUG^c^, BOTMP, Goal Attainment Scale, Strengths and Difficulties Questionnaire	Potential therapeutic benefit
Baque et al [36]	Mitii program	30 min/day, 6 days a week for 20 weeks	Mitii program from 12 available modules including (1) gross motor, (2) combined cognitive and visual perception, and (3) upper limb activities. Gross motor activities included sit-to-stands, squats, lunges, aerobic and balance tasks. Daily programs comprised of approximately 40% gross motor and 60% cognitive-upper limb modules.	Week 0 and week 20	30-second, repetition maximum functional strength tests for the lower limb; 6-minute walk test; high-level Mobility Assessment Tool; TUG; habitual physical activity	Functional strength improved in the treatment group.
Lai et al [37]	M2M^d^ program	3 times per week for 4 weeks	M2M included videos that participants were asked to complete 3 times each week at home. In week 1, the prescription generally included a total of 48 minutes of video time, which included a mixed range of motion exercise routines with guided instructions. In weeks 2 and 3, the patients were prescribed 70 minutes of video time. Week 2 included 2 mix ed range-of-motion and muscle-strengthening routines. Week 3 included 2 range of motion routines and 1 strengthening routine. In week 4, participants were prescribed 100 minutes of video time, which included 2 range-of-motion exercise routines, 1 for strength, and 1 for cardiovascular exercise.	Pre- and posttraining	LTPA^e^, pain, and fatigue	LTPA improved in the treatment group.
Sakzewski et al [38]	Mitii program	30 min/day, 6 days a week for 20 weeks	11 available modules in Mitii with 60% targeting combined cognitive, visual perceptual, and upper limb activities, and 40% focused on gross motor activities.	Week 0 and week 20	AMPS^f^, Melbourne Assessment of Unilateral Upper Limb Function, Jebsen–Taylor Test of Hand Function, Test of Visual Perceptual Skills, Assisting Hand Assessment, COPM^g^	Negligible changes
Tianming [39]	Remote home rehabilitation	More than 1.5 hours per day, 5 days per week for 12 weeks	According to the limb function of children, combined with the demands of children and parents, and according to their home environment, interests, the number of children under care and other factors, an individualized remote home rehabilitation training plan was formulated. For spastic disease, all training items were filmed in the whole process and participants were equipped with written materials to explain the training plan and training actions. A training scale was developed that included the training time, frequency, and cooperation status of children. Video rehabilitation.	Pre- and posttraining	GMFM-66^h^, balance function, spasticity degree of hamstring and gastrocnemius on the affected side, measurement of ankle joint range of motion	The motor function, balance function, ankle joint range of motion, and muscle tension of the treatment group were significantly improved.
M Piovesana et al [40]	Mitii program	20-30 min/day, 6 days a week for 20 weeks	Mitii consists of upper limb, cognitive, visual perceptual and physical activity training. Selected from 14 training modules. The training modules include approximately 60% visual perceptual, upper limb and cognitive games, and 40% physical activity games.	Pre- and posttraining	Attentional control, cognitive flexibility, goal setting, information processing; EF^i^ performance was assessed via parent report	Mitii has potential to intervene in executive function.
Mitchell et al [41]	Mitii program	30 min/day, 6 days a week for 20 weeks	Each program was set up such that physical activity games were interspersed with upper limb and visual perceptual games. Gross motor exercises comprised approximately 40% of the overall program. The intensities of lower-limb strength exercises for week 1 was determined by setting tasks at approximately 75% of repetition maximum determined during baseline assessments. On average, week 1 started with 7 activities of between 5 repetitions and 10 repetitions lasting approximately 60 seconds per activity, and progressed to 11 games of up to 20 repetitions lasting approximately 90 seconds with the addition of step blocks and balance foam.	Pre- and posttraining	Maximal repetitions of functional strength tasks, 6-minute walk test	Functional strength and walking endurance improved in the treatment group.
Pin and Butler [42]	Interactive computer play training	20 min/day, 4 times per week for 6 weeks	All children played the computer game sitting with hips and knees at 90° and both feet supported. The game was started at a medium-low level of difficulty. If the child was unable to score for 3 consecutive trials, the difficulty level was reduced by 1 level. Similarly, if the child scored full points for 3 consecutive trials, the difficulty level was raised by 1 level.	Week 0, week 3, week 6, week 12	Pediatric Reach Test, GMFM-66, 2-Minute Walk Test	A 6-week interactive computer play training was feasible and safe for children with moderate cerebral palsy.
Piovesana et al [43]	Mitii program	20-30 min/day, 6 days a week for 20 weeks	Therapists selected from the 11 available Mitii modules which included modules targeted to (1) gross motor or physical activity (eg, repetitive star jumps), (2) combined cognitive and visual perception (eg, match the concepts), or (3) upper limb (eg, moving upper limb to solve a mathematical problem). Modules were selected for an individualized program time of 30 minutes. Programs were designed to include 40% gross motor and 60% cognitive-upper limb training modules.	Pre- and posttraining	Attentional control, cognitive flexibility, goal setting, and information processing; Delis-Kaplan Executive Functioning System; Comprehensive Trail Making Test; Tower of London; Test of Everyday Attention for Children	There is no additional benefit compared to standard care.
Sabel et al [44]	Active video gaming	30 min/day, at least 5 days a week for 10-12 weeks	The Nintendo Wii is controlled by one or 2 hand-held remote controls and requires movement to play the games. Each child received 2 pairs of controls, which enabled them to play with friends, and a balance board, the Wii Fit. As the intensity of the games varied, participants were instructed to start every session with a physically more demanding game for at least 10 min, before considering switching to a slower-paced game, such as a balance game.	Pre- and posttraining	Gaming time and compliance, physical activity levels, physical functioning	Physical coordination improved in the treatment group.
Sabel et al [45]	Active video gaming	30 min/day, at least 5 days a week for 10-12 weeks	Nintendo Wii, an off-the-shelf, motion-controlled video console, was used for home-based physical exercise during the intervention period, for a minimum of 30 minutes per day, at least 5 days per week, for 10 to 12 weeks. Games were chosen to mainly stimulate physical activity but also included less physically demanding games, such as balance games, using the Wii Fit balance board accessory.	Pre- and posttraining	AMPS, cognitive assessment, execution of activities of daily living	Motor and process skills in activities of daily living improved in the treatment group.
James et al [46]	Mitii program	20-30 min/day, 6 days per week for 20 weeks	Therapists selected from 14 training modules to devise a program that included approximately 60% cognitive or visual perceptual activities combined with upper limb (predominantly the impaired upper limb), and 40% gross motor activities.	Pre- and posttraining	AMPS, assisting hand assessment, JTTHF^j^, Melbourne Assessment of Unilateral Upper Limb Function, COPM, TVPS-3^k^	AMPS, JTTHF dominant upper limb, COPM, and TVPS-3 improved in the treatment group.
Wang et al [47]	CIT^l^ plus Wii-augmented CIT	2.25 hours per session and 2 sessions per week for 8 weeks	To elicit various motor skills, multiple games were used (eg, Wii Sports Resort, Wii Sports, Mario Sports Mix, Cooking Mama: Cook Off, Let’s Tap, and Happy Dance Collection).	Week 0, week 4, and week 8	BOTMP, Revised Pediatric Motor Activity Log, ABILHAND-Kids, Functional Independence Measures for Children, Parenting Stress Index–Short Form, Test of Playfulness, Engagement Questionnaire	CIT-Wii yields no significant difference in treatment effects from conventional CIT and may provide psychosocial benefits.

^a^BOTMP: Bruininks-Oseretsky Test of Motor Proficiency.

^b^WM: working memory.

^c^TUG: Timed up and Go test.

^d^M2M: Movement-to-Music.

^e^LTPA: leisure-time physical activity.

^f^AMPS: Assessment of Motor and Process Skills.

^g^COPM: Canadian Occupational Performance Measure.

^h^GMFM-66: Gross Motor Function Measure-66.

^i^EF: executive function.

^j^JTTHF: Jebsen–Taylor Test of Hand Function.

^k^TVPS-3: Test of Visual Perceptual Skills.

^l^CIT: constraint-induced therapy.

A total of 10 studies [31,35,36,38,39,42,44-47] assessed motor function, 5 studies [36,37,41,42,44] assessed physical activity level, 3 studies [31,36,41] assessed lower limb strength, 5 studies [34,38,40,43,46] assessed visual processing skills, 3 studies [33,34,40] assessed working memory, 3 studies [33,40,43] assessed attention, 2 studies [40,43] assessed processing speed, 4 studies [33,34,40,43] assessed executive function, 2 studies [38,46] assessed hand function, 4 studies [32,38,46,47] assessed upper limb function, 4 studies [31,35,39,42] assessed balance function, and 2 studies [38,46] assessed occupational performance.

### Risk of Bias

**Figure 2:**
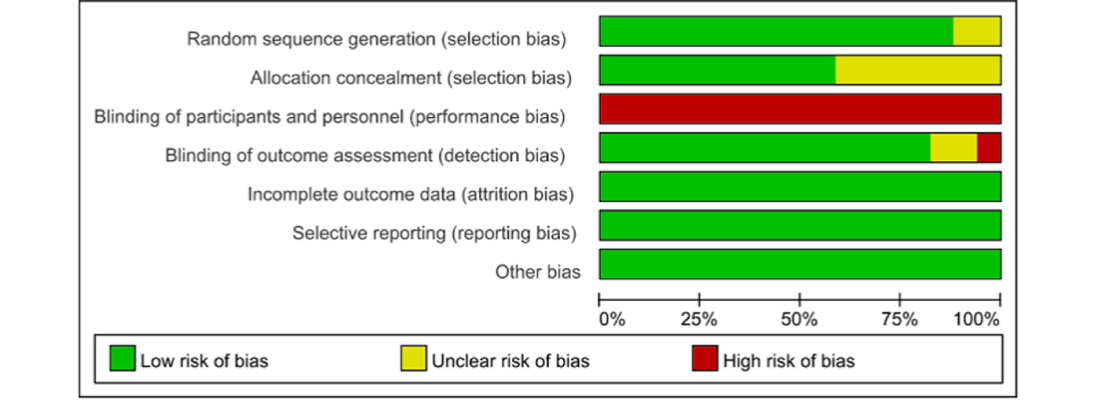
Overall risk of bias assessment results.

**Figure 3:**
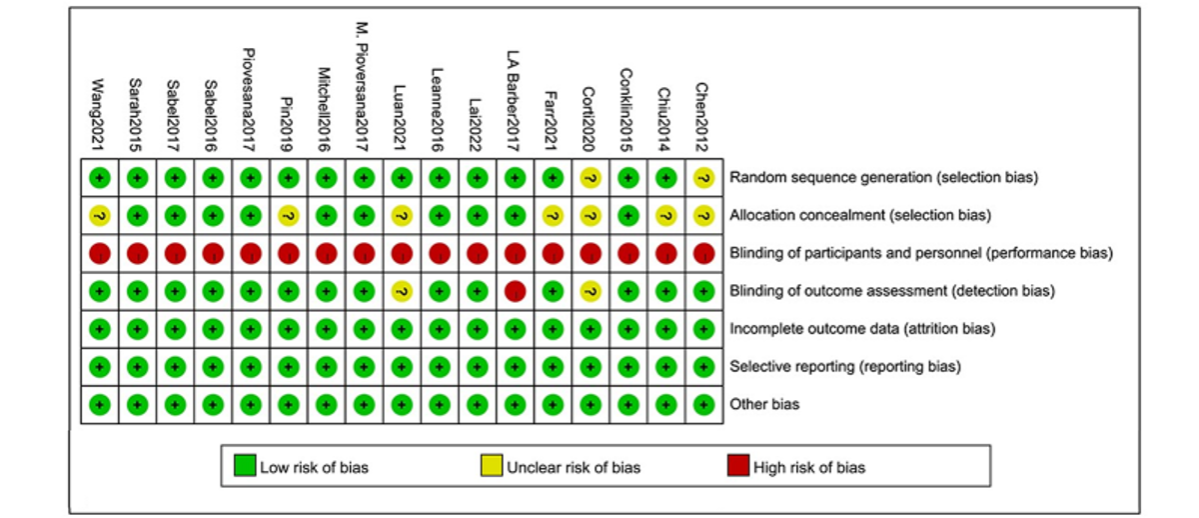
The results of bias risk assessment of each study. Green symbols indicate low risk of bias, yellow symbols indicate unclear risk of bias, and red symbols indicate high risk of bias.

Figures 2 and 3 [31-35,37,41-45,47] show the risk of bias assessments. We categorized high, low, or unclear risk of bias for all 17 included studies [31-47]. The greatest risk of bias was due to the blinding of participants and personnel, because the nature of rehabilitation physical activity interventions makes it difficult to blind patients or health care providers. One study [36] had a high risk of blinding outcome assessment in unblinded outcome assessors. There was no high risk of bias in the randomized sequence generation, allocation concealment, incomplete outcome data, or selective reporting.

After summarizing the assessments across items in the tool for each outcome within each trial, the results showed that motor function, physical activity level, lower limb strength, visual processing skills, letter-number sequencing, arithmetic calculation, working memory, attention, coding, symbol search, cognitive flexibility, executive function, upper limb and hand function, balance function, and occupational performance were at a high risk of bias within and across trials (Multimedia Appendix 2).

### Results of the Meta-Analysis

#### Effect on Motor Function

We included 10 studies (377 individuals) [31,35,36,38,39,42,44-47] in the meta-analysis. Regarding motor function, 4 studies used the BOTMP scale [48], 2 studies used the GMFM-66 scale, and 4 studies used the AMPS motor skills.

**Figure 4:**
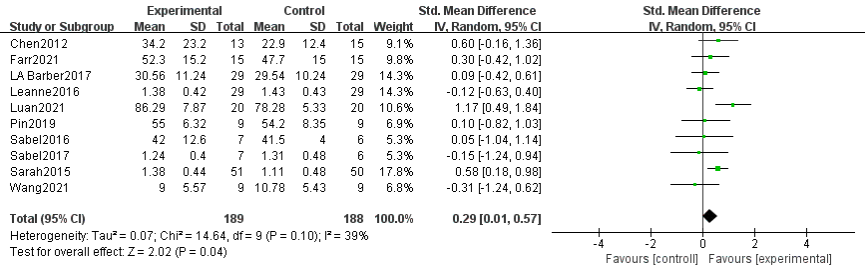
Effects of web-based telerehabilitation programs on motor function in children and adolescents with brain injury.

The results showed that there were significant differences between the treatment and control groups, and the treatment group had better motor function than the control group with insignificant heterogeneity (standardized mean difference [SMD] 0.29, 95% CI 0.01-0.57, *P*=.04; heterogeneity: *I*^2^ 39%; *P*=.10; Figure 4 [31,35,36,38,39,42,44-47]).

To better evaluate the results, subgroup analysis of motor function was performed according to scoring scales, intervention duration, interaction with medical professionals, test sample size, and nation. However, there was no significant difference in the subgroup analysis of motor function (Multimedia Appendix 3).

#### Effect on Physical Activity Level

**Figure 5:**
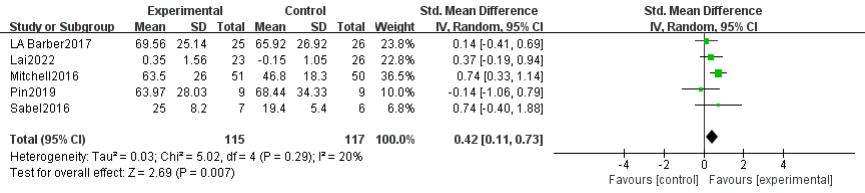
Effects of web-based telerehabilitation programs on physical activity level in children and adolescents with brain injury.

A total of 5 studies (232 individuals) measured physical activity levels, 2 of which [36,41] used functional strength composite training, 1 [37] used leisure-time physical activity, 1 [44] used BOTMP, and another study [42] used 2-minute walk test to measure physical activity levels. The results showed that the intervention group based on web therapy had significantly better physical activity levels than the control group with insignificant heterogeneity (SMD 0.42, 95% CI 0.11-0.73, *P*=.007; heterogeneity: *I*^2^=20%; *P*=.29; Figure 5 [36,37,41,42,44]).

#### Effect on Lower Limb Strength

**Figure 6:**
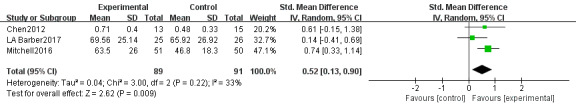
Effects of web-based telerehabilitation programs on lower limb strength in children and adolescents with brain injury.

A total of 3 studies (180 individuals) [31,36,41] explored the effects of web-based telerehabilitation programs on lower extremity strength. These results were pooled to show a significant improvement in lower extremity strength in the treatment group with insignificant heterogeneity (SMD 0.52, 95% CI 0.13-0.90, *P*=.009; heterogeneity: *I*^2^=33%; *P*=.22; Figure 6 [31,36,41]).

#### Effect on Visual Processing Skills

**Figure 7:**
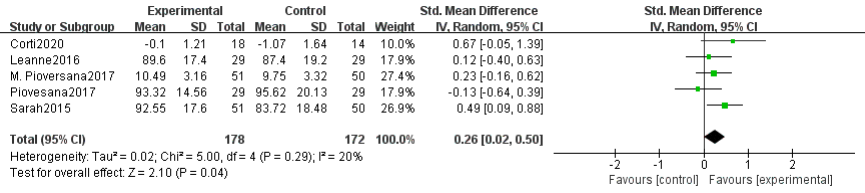
Effects of web-based telerehabilitation programs on visual processing skills in children and adolescents with brain injury.

The results of the 5 included studies (350 individuals) [34,38,40,43,46] showed that there were significant differences between the treatment and control groups and that the treatment group performed better than the control group by performing web-based telerehabilitation training at home (SMD 0.26, 95% CI 0.02-0.50, *P*=.04; heterogeneity: *I*^2^=20%; *P*=.29; Figure 7 [34,38,40,43,46]).

#### Effect on Executive Function

As executive function encompasses multiple aspects, this study analyzed working memory, attention, processing speed, cognitive flexibility, and executive function.

Working memory was assessed using the Woodcook-Johnson III Achievement Test, which includes letter-number sequencing, arithmetic calculation, and working memory. Two studies [33,40] assessed letter-number sequencing and showed that the treatment group had better letter-number sequencing than the control group with no heterogeneity (SMD 1.26, 95% CI 0.26-2.26; *P*=.01; heterogeneity: *I*^2^=0%; *P*=.92). Two studies [33,34] assessed arithmetic calculation and showed no significant difference in arithmetic calculation between the treatment and control groups (SMD=0.46, 95% CI −0.73 to 1.65, *P*=.45; heterogeneity: *I*^2^=0%, *P*=.85). Two studies [33,34] assessed working memory and showed no significant difference in working memory between the treatment and control groups (SMD 3.59, 95% CI −3.10 to 10.27, *P*=.29; heterogeneity: *I*^2^=73%, *P*=.05).

Three studies (221 individuals) [33,40,43] used the Digit Span Test or Children’s Daily Attention Test to assess attention function. The results showed a significant increase in attention in the treatment group compared to the control group, accompanied by insignificant statistical heterogeneity (SMD 0.38, 95% CI 0.09-0.66, *P*=.009; heterogeneity *I*^2^=9%, *P*=.33).

Processing speed was assessed using the Wechsler Individual Achievement Test, which contains coding and symbol searches. Two studies [40,43] assessing coding showed no significant difference in coding between the treatment and control groups (SMD 0.63, 95% CI −0.63 to 1.90; *P*=.32; heterogeneity: *I*^2^=40%; *P*=.20). Two studies [40,43] assessed symbol search, and the treatment group had a better symbol search than the control group (SMD 1.18, 95% CI 0.43-1.93; *P*=.002; heterogeneity: *I*^2^=0%; *P*=.52). Four studies (253 individuals) [33,34,40,43] used the Behavior Rating Inventory of Executive Function to assess cognitive flexibility, and 3 studies (221 individuals) [33,40,43] assessed executive function. However, the results showed that there was no statistically significant difference between the treatment and control groups (SMD −0.69, 95% CI −3.06 to 1.69; *P*=.57; heterogeneity: *I*^2^=40%; *P*=.17; SMD −0.25, 95% CI −0.52 to 0.01, *P*=.06; heterogeneity: *I*^2^=0%, *P*=.76).

The summary results are shown in Table 3, and the forest plots are shown in Multimedia Appendix 4 [33,34,40,43].

**Table 3:** Summary of executive function.

Result	Number of studies included	SMD^a^ (95% CI)	*P* value	Heterogeneity	
				*I*^2^, %	*P* value
Letter-number sequencing	2	1.26 (0.26 to 2.26)	.01	0	.92
Arithmetic calculation	2	0.46 (–0.73 to 1.65)	.45	0	.85
Working memory	2	3.59 (–3.10 to 10.27)	.29	73	.05
Attention	3	0.38 (0.09 to 0.66)	.009	9	.33
Coding	2	0.63 (–0.63 to 1.90)	.32	40	.20
Symbol search	2	1.18 (0.43 to 1.93)	.002	0	.52
Cognitive flexibility	4	−0.69 (–3.06 to 1.69)	.57	40	.17
Executive function	3	−0.25 (–0.52 to 0.01)	.06	0	.76

^a^SMD: standardized mean difference.

#### Effect on Other Outcome Measures

For hand and upper limb function, 4 studies (237 individuals) [32,38,46,47] and 2 studies (159 individuals) [38,46] evaluated whether web-based telerehabilitation programs had an impact on these functions. The results showed no significant difference between the treatment and control groups (SMD −0.08, 95% CI −0.33 to 0.18; *P*=.55; heterogeneity: *I*^2^=0%; *P*=.96; SMD 1.37, 95% CI −3.28 to 6.02; *P*=.56; heterogeneity: *I*^2^=0%; *P*=.67).

The results of the 4 included studies (107 individuals) [31,35,39,42] showed no significant difference in balance between the treatment and control groups (SMD 0.45, 95% CI −0.15 to 1.06; *P*=.14; heterogeneity: *I*^2^=57%; *P*=.07). Two studies (159 individuals) [38,46] assessed occupational function using the COPM, and the results showed no significant differences between the treatment and control groups (SMD 0.51, 95% CI −1.04 to 2.06; *P*=.52; heterogeneity: *I*^2^=90%; *P*=.002).

The summary results are shown in Table 4, and the forest plots are shown in Multimedia Appendix 5 [31,32,35,38,39,42,46,47].

**Table 4:** Summary of other outcomes.

Result	Number of studies included	SMD^a^ (95% CI)	*P* value	Heterogeneity	
				*I*^2^, %	*P* value
Hand function	4	−0.08 (−0.33 to 0.18)	.55	0	.96
Upper limb function	2	1.37 (−3.28 to 6.02)	.56	0	.67
Balance function	4	0.45 (−0.15 to 1.06)	.14	57	.07
COPM^b^	2	0.51 (−1.04 to 2.06)	.52	90	.002

^a^SMD: standardized mean difference.

^b^COPM: Canadian Occupational Performance Measure.

### Sensitivity Analyses

Galbraith plots were drawn and sensitivity analyses were performed using methods that removed individual studies to determine the reasons for heterogeneity in balance outcomes. Through the Galbraith plot, we found that the study by Luan et al [39] intersects the 95% CI regression line, suggesting that this may be the source of the heterogeneity anomaly (Multimedia Appendix 6 [31,35,39,42]). After the removal of the study by Luan et al [39], the outcome of balance function was still not statistically significant (SMD 0.17, 95% CI −0.31 to 0.66; *P*=.48), but there was a statistically significant decrease in heterogeneity (*I*^2^=0%; *P*=.57). No Galbraith plots were created for the COPM outcome because only 2 studies were included.

### Certainty of Evidence

GRADEpro GDT was used to evaluate the quality of evidence. The level of evidence was initially “high” because most of the included studies were RCTs (17/17, 100%). Because the nature of rehabilitation makes blinding of participants and health care providers difficult, all studies have one domain that is at high risk of bias, which means that all studies are at high risk of bias. It is sufficient to affect the interpretation of results, so it has to be downgraded by 2 levels for the risk of bias for all outcomes. Moderate inconsistencies in balance function (*I*^2^=57%) and large inconsistencies in working memory and occupational performance (*I*^2^=73% and *I*^2^=90%), so the quality of inconsistencies for balance function was downgraded by 1 level, and for working memory and occupational performance were downgraded by 2 levels. All outcomes were assessed using standardized scales; therefore, the quality of indirectness for all the results was not downgraded. As the pooled effect sizes of the 95% CI for arithmetic calculation, working memory, coding, cognitive flexibility, executive function, hand and upper limb function, balance function, and occupational performance contained null values, the quality of evidence for these results was downgraded by 1 level of imprecision. Egger test for publication bias was conducted for the meta-analysis of motor function. The results showed that the *P* value was insignificant (*P*=.21), and the funnel plot appeared asymmetrical (Multimedia Appendix 7). Thus, publication bias was unlikely; hence, the quality of evidence was not downgraded because of the absence of publication bias.

In summary, the quality was moderate in the outcome of motor function. The outcomes of physical activity level, lower extremity strength, visual processing skills, letter-number sequencing, attention, and symbol search were low quality. Arithmetic calculations, working memory, coding, cognitive flexibility, executive function, hand and upper limb function, balance function, and occupational performance were of very low quality (Multimedia Appendix 8).

## Discussion

### Principal Findings

A total of 17 RCTs on the effects of web-based telerehabilitation programs on children and adolescents with ABI were included. The results showed that in the primary outcome of motor function, as assessed using the GMFM-66, BOTMP, and AMPS, the treatment group showed better improvement than the usual rehabilitation group.

Regarding secondary outcomes, the physical activity level and lower limb strength of children and adolescents with ABI significantly improved, which is consistent with the improvement in motor function. The visual performance of children and adolescents with ABI in the treatment group was better than that in the control group, which may be due to the enrichment of spatial items and bright colors of visual stimuli in these game training modules, and the children and adolescents’ visual discrimination and visual sequential memory abilities were improved [49,50].

However, the outcomes with no statistically significant results, such as working memory, executive ability, cognitive flexibility, balance function, hand and upper limb function, and COPM, may be due to the small sample size, large differences in sample characteristics, or the inclusion of data from only 1 or 2 studies, which could not evaluate the overall training results. The types of brain injury and intervention programs differed between the included studies, and these interventions were heterogeneous in nature. Therefore, further studies are needed to explain the results of this systematic review and meta-analysis.

### Discussion of Subgroup Analyses

In the inclusion of the studies, 10 studies contained the results of motor function. After subgroup analyses according to scoring scales, intervention duration, interaction with medical professionals, test sample size, and nation, the results were not significant, which may be due to the small number of studies included and the small sample size in each subgroup; thus, there was a nonsignificant effect size.

### Advantages of Web-Based Intervention

For motor function, the interventions included in the literature included Wii Fit computer games, Mitii training programs, and home-based virtual bicycle training, which are more novel, interesting, and attractive to children and adolescents. Once weekly, remote supervision [39] allowed therapists to adjust the training plan in a timely manner according to the training effect of children and adolescents, increase the difficulty of training in a stepwise manner, and ensure the normal progress of the intervention. Because the training was performed at home, the children’s and adolescents’ vigilance was lower, and they were more inclined to participate in the training in the company of their parents, which reduced the possibility of children and adolescents being nervous and afraid to participate in the training in the hospital and alleviated the training effect. Movement disorders in children and adolescents with brain injuries often require effective long-term intervention [40]. Therefore, it is important for children and adolescents to be actively involved in the treatment. Otherwise, secondary injury caused by insufficient physical activity will cause further damage and lead to more serious consequences [40], such as decreased muscle strength or psychological problems [40,51].

For secondary outcomes, children and adolescents with ABI showed improvements in physical activity level, lower limb strength, letter-number sequencing, attention, and symbol search with unimportant heterogeneity and more precise estimates of cognitive domains compared with previous meta-analyses [15].

### Limitations

This study has several limitations. First, only English literature was included, which may have missed relevant studies. Second, 5 databases, including PubMed, Web of Science, Embase, Scopus, and Cochrane Library, were searched; however, the search platform may not be sufficiently comprehensive, and some articles may have been overlooked. Third, owing to the nature of rehabilitation treatment, it was impossible to blind the participants and therapists. Some studies did not explain whether the evaluators were blinded and how the allocation was concealed, and there may have been a blinding bias. Fourth, there was a certain degree of heterogeneity in the different population categories and exercise prescriptions included in the study. Fifth, because part of the data were the result of the questionnaire surveys, there may be subjective bias. Sixth, no separate analysis was performed for the untreated and routine care groups. Finally, some studies had follow-up data, but the intervention effect was not obvious after the follow-up, which may be due to the short intervention time or other factors. Therefore, it is difficult to evaluate long-term effects.

### Future Directions

Future studies should search more databases, include more populations, study the effects of web-based telerehabilitation programs on children and adolescents with different types of brain injuries, and collect more follow-up data on long-term rehabilitation.

### Conclusions

In conclusion, this study showed that web-based telerehabilitation programs helped improve motor function, physical performance, lower limb strength, letter-number sequencing, attention, symbol search, and visual processing skills in children and adolescents with ABI. Web-based telerehabilitation interventions provided great convenience for children and adolescents with ABI who need long-term treatment, and the widespread implementation of remote interventions also provided children and adolescents in remote areas where rehabilitation services are harder to access the opportunity to obtain the treatment they needed. Overall, web-based telerehabilitation programs are safe, effective, and enjoyable physical therapy methods. Future studies could expand the sample size according to the type of disease and increase the follow-up time according to different exercise prescriptions to further refine the long-term effects of this intervention on various functions in children and adolescents with ABI.

## Appendix

Multimedia Appendix 1 Search strategy.

Multimedia Appendix 2 Summary of the assessment of risk of bias for each outcome within and across trials.

Multimedia Appendix 3 Results of subgroup analysis of motor function.

Multimedia Appendix 4 Summary of forest plots for executive function.

Multimedia Appendix 5 Summary of forest plots for other outcomes.

Multimedia Appendix 6 Galbraith plot of the balance outcome.

Multimedia Appendix 7 Egger test for motor function.

Multimedia Appendix 8 Grading of Recommendations Assessment, Development, and Evaluation quality evaluation of the outcomes.

Multimedia Appendix 9 PRISMA 2020 Checklist.

## Abbreviations

ABI: acquired brain injury
AMPS: Assessment of Motor and Process Skills
BOTMP: Bruininks-Oseretsky Test of Motor Proficiency
COPM: Canadian Occupational Performance Measure
GMFM-66: Gross Motor Function Measure-66
GRADEpro GDT: Grading of Recommendations Assessment, Development, and Evaluation
PRISMA: Preferred Reporting Items for Systematic Reviews and Meta-Analyses
RCT: randomized controlled trial
SMD: standardized mean difference

## Data Availability

All data generated or analyzed during this study are included in the published article. All figures have been published in an open access journal.
