# Supplementary material for: The Effect of Web-Based Telerehabilitation Programs on Children and Adolescents With Brain Injury: Systematic Review and Meta-Analysis
Source: J Med Internet Res. 2023 Dec 25;25:e46957. doi: 10.2196/46957 (PMC10775025; doi:10.2196/46957)
Supplement: Multimedia Appendix 1 [file jmir_v25i1e46957_app1.docx]

**Multimedia Appendix 1**

**Search strategy**

**This Supplementary file provides the search strategy details, performed December 3rd, 2022**

| Datebase name | Search strategies: key words and how these  were combined in the search | Laster  update | Number of  studies  identified |
| --- | --- | --- | --- |
| PubMed | ((((((((((((((((Telerehabilitation[MeSH Terms]) OR (Telerehabilitations[Title/Abstract])) OR (Tele-rehabilitation[Title/Abstract])) OR (Tele rehabilitation[Title/Abstract])) OR (Tele-rehabilitations[Title/Abstract])) OR (Remote Rehabilitation[Title/Abstract])) OR (Rehabilitation, Remote[Title/Abstract])) OR (Rehabilitations, Remote[Title/Abstract])) OR (Remote Rehabilitations[Title/Abstract])) OR (Virtual Rehabilitation[Title/Abstract])) OR (Rehabilitation, Virtual[Title/Abstract])) OR (Rehabilitations, Virtual[Title/Abstract])) OR (Virtual Rehabilitations[Title/Abstract])) OR (web)) OR (home)) AND ((((((((brain) OR (cranial)) OR (cerebral)) OR (head)) OR (intracranial)) OR (fossa)) OR (subarachnoid)) OR (cerebrocranial))) AND (((children) OR (pediatric)) OR (adolescents)) | 3rd  December  2022 | 9530 |
| Scopus | #1 ( TITLE-ABS-KEY ( brain ) OR TITLE-ABS-KEY ( cranial ) OR TITLE-ABS-KEY ( cerebral ) OR TITLE-ABS-KEY ( head ) OR TITLE-ABS-KEY ( intracranial ) OR TITLE-ABS-KEY ( fossa ) OR TITLE-ABS-KEY ( subarachnoid ) OR TITLE-ABS-KEY ( cerebrocranial )  3,530,000  #2 ( TITLE-ABS-KEY ( children ) OR TITLE-ABS-KEY ( pediatric ) OR TITLE-ABS-KEY ( adolescents ) )  4,853,403  #3 ( TITLE-ABS-KEY ( telerehabilitation ) OR TITLE-ABS-KEY ( telerehabilitations ) OR TITLE-ABS-KEY ( tele-rehabilitation ) OR TITLE-ABS-KEY ( tele AND rehabilitation ) OR TITLE-ABS-KEY ( tele-rehabilitations ) OR TITLE-ABS-KEY ( remote AND rehabilitation ) OR TITLE-ABS-KEY ( rehabilitation, AND remote ) OR TITLE-ABS-KEY ( rehabilitations, AND remote ) OR TITLE-ABS-KEY ( remote AND rehabilitations ) OR TITLE-ABS-KEY ( virtual AND rehabilitation ) OR TITLE-ABS-KEY ( rehabilitation, AND virtual ) OR TITLE-ABS-KEY ( rehabilitations, AND virtual ) OR TITLE-ABS-KEY ( virtual AND rehabilitations ) OR TITLE-ABS-KEY ( web ) OR TITLE-ABS-KEY ( home ) )  1,386,138  #4 #3 AND #2 AND #1 9406 | 3rd  December  2022 | 9406 |
| Web of  science | #1 brain (Topic) or cranial (Topic) or cerebral (Topic) or head (Topic) or intracranial (Topic) or fossa (Topic) or subarachnoid (Topic) or cerebrocranial (Topic) 3,578,721  #2 children (Topic) or pediatric (Topic) or adolescents (Topic) 5,192,878  #3 telerehabilitation (Topic) or telerehabilitations (Topic) or tele-rehabilitation (Topic) or tele-rehabilitations (Topic) or remote rehabilitation (Topic) or remote rehabilitations (Topic) or rehabilitation, remote (Topic) or virtual rehabilitation (Topic) or virtual rehabilitations (Topic) or web (Topic) or home (Topic) 1,200,953  #4 #3 AND #2 AND #1 10638 | 3rd  December  2022 | 10638 |
| Cochrane  library | #1 (Brain):ti,ab,kw OR (cranial):ti,ab,kw OR (head):ti,ab,kw OR (cerebral):ti,ab,kw OR (intracranial):ti,ab,kw OR (fossa):ti,ab,kw OR (subarachnoid):ti,ab,kw OR (cerebrocranial):ti,ab,kw 122,973  #2 (children):ti,ab,kw OR (pediatric):ti,ab,kw OR (adolescents):ti,ab,kw 187,960  #3 (telerehabilitation):ti,ab,kw OR (telerehabilitations):ti,ab,kw OR (tele-rehabilitation):ti,ab,kw OR (tele-rehabilitations):ti,ab,kw OR (remote rehabilitation):ti,ab,kw OR (rehabilitation, remote):ti,ab,kw OR (remote rehabilitations):ti,ab,kw OR (virtual rehabilitation):ti,ab,kw OR (virtual rehabilitations):ti,ab,kw OR (web):ti,ab,kw OR (home):ti,ab,kw 68,450  #4 #3 AND #2 AND #1 1382 | 3rd  December  2022 | 1382 |
| Embase | #1 'brain'/exp OR brain OR head OR cranial OR cerebral OR intracranial OR fossa OR subarachnoid OR cerebrocranial 4,092,860  #2 'children'/exp OR children OR pediatric OR adolescents 4,412,168  #3 'telerehabilitation'/exp OR telerehabilitation OR telerehabilitations:ab,ti OR 'tele rehabilitation':ab,ti OR 'tele rehabilitations':ab,ti OR 'remote rehabilitation':ab,ti OR 'rehabilitation, remote':ab,ti OR 'rehabilitations, remote':ab,ti OR 'remote rehabilitations':ab,ti OR 'virtual rehabilitation':ab,ti OR 'rehabilitation, virtual':ab,ti OR 'rehabilitations, virtual':ab,ti OR 'virtual rehabilitations':ab,ti OR web OR home  1,070,299  #4 #3 AND #2 AND #1 21,066 | 3rd  December  2022 | 21066 |
