# Supplementary material for: The Effect of Web-Based Telerehabilitation Programs on Children and Adolescents With Brain Injury: Systematic Review and Meta-Analysis
Source: J Med Internet Res. 2023 Dec 25;25:e46957. doi: 10.2196/46957 (PMC10775025; doi:10.2196/46957)
Supplement: Multimedia Appendix 2 [file jmir_v25i1e46957_app2.docx]

**Multimedia Appendix 2**

**Summary of the assessment of the risk of bias for each outcome within and across trials**

| **Outcome** | **Within a trial** | **Across trials** |
| --- | --- | --- |
| Motor function | High risk of bias | High risk of bias |
| Physical activity level | High risk of bias | High risk of bias |
| Lower limb strength | High risk of bias | High risk of bias |
| Visual processing skills  Letter-number sequencing  Arithmetic calculation | High risk of bias  High risk of bias  High risk of bias | High risk of bias  High risk of bias  High risk of bias |
| Working memory | High risk of bias | High risk of bias |
| Attention | High risk of bias | High risk of bias |
| Coding  Symbol search | High risk of bias  High risk of bias | High risk of bias  High risk of bias |
| Cognitive flexibility | High risk of bias | High risk of bias |
| Executive function | High risk of bias | High risk of bias |
| Hand function | High risk of bias | High risk of bias |
| Upper limb function | High risk of bias | High risk of bias |
| Balance function | High risk of bias | High risk of bias |
| Occupational performance | High risk of bias | High risk of bias |
