# Supplementary material for: The Effect of Web-Based Telerehabilitation Programs on Children and Adolescents With Brain Injury: Systematic Review and Meta-Analysis
Source: J Med Internet Res. 2023 Dec 25;25:e46957. doi: 10.2196/46957 (PMC10775025; doi:10.2196/46957)
Supplement: Multimedia Appendix 3 [file jmir_v25i1e46957_app3.docx]

**Multimedia Appendix 3**

**Results of subgroup analysis of motor function**

|  |
| --- |

| **Groups** | **n** | **SMD** | **95%CI** | ***I²*（%）** | ***P*** |
| --- | --- | --- | --- | --- | --- |
| **Scoring scale** |  |  |  |  |  |
| GMFM | 2 | 0.68 | (-0.36,1.72) | 70.0 | .20 |
| AMPS motor | 4 | 0.18 | (-0.19,0.56) | 45.0 | .34 |
| BOTPM | 4 | 0.23 | (-0.19,0.65) | 0.0 | .29 |
| **Intervention duration** |  |  |  |  |  |
| <12 weeks | 7 | 0.34 | (-0.06,0.74) | 35.0 | .10 |
| ≥12 weeks | 3 | 0.21 | (-0.22,0.64) | 60.0 | .33 |
| **Interact with medical professionals** |  |  |  |  |  |
| Interaction | 8 | 0.20 | (-0.04,0.45) | 11.0 | .11 |
| no interaction | 2 | 0.74 | (-0.11,1.60) | 67.0 | .39 |
| **Sample size** |  |  |  |  |  |
| <50 | 7 | 0.34 | (-0.06,0.74) | 35.0 | .10 |
| ≥50 | 3 | 0.21 | (-0.22,0.64) | 60.0 | .33 |
| **Nation** |  |  |  |  |  |
| Asian | 4 | 0.45 | (-0.19,1.09) | 59.0 | .17 |
| Europe | 3 | 0.14 | (-0.39,0.66) | 0.0 | .61 |
| Oceania | 3 | 0.21 | (-0.22,0.64) | 60.0 | .33 |
