# Supplementary material for: The Effect of Web-Based Telerehabilitation Programs on Children and Adolescents With Brain Injury: Systematic Review and Meta-Analysis
Source: J Med Internet Res. 2023 Dec 25;25:e46957. doi: 10.2196/46957 (PMC10775025; doi:10.2196/46957)

**Multimedia Appendix 4**

**Summary of forest plots for executive function**

1. **Forest plot of effects on working memory[33,34,40]**


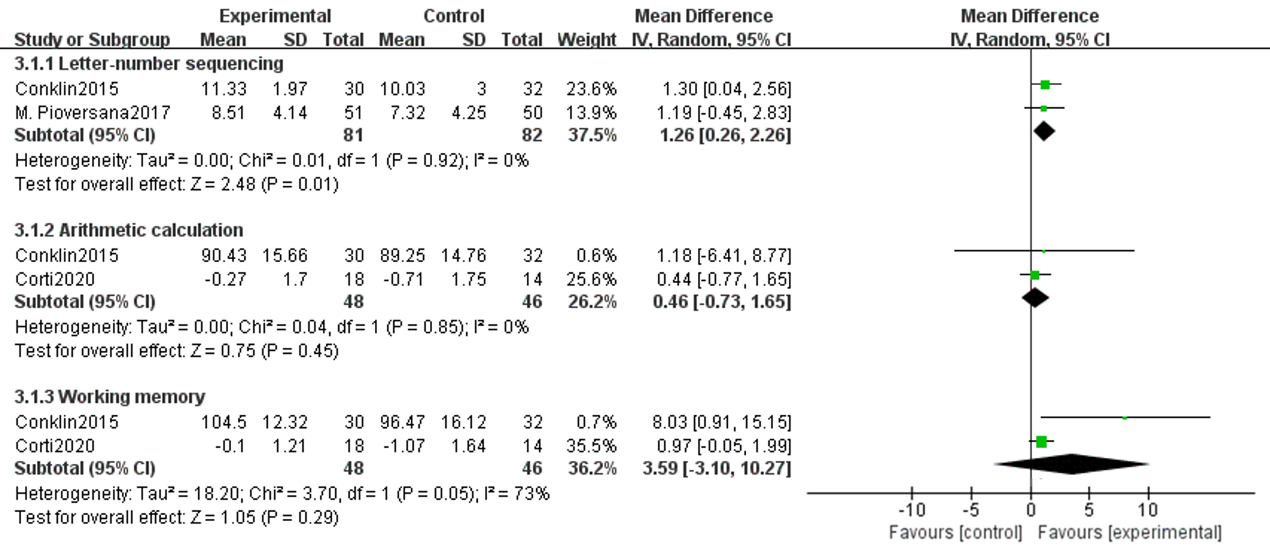


1. **Forest plot of effects on attention[33,40,43]**


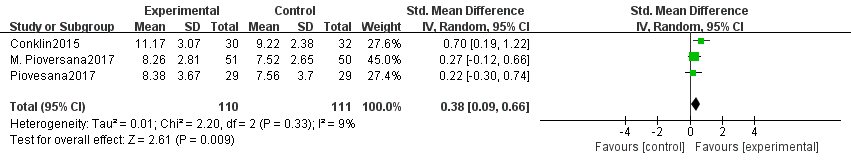


1. **Forest plot of effects on processing speed[40,43]**


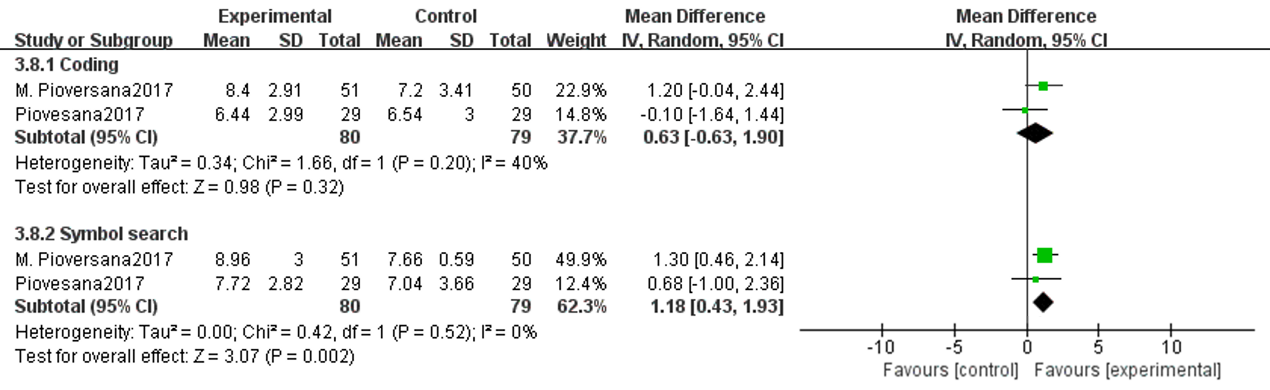


1. **Forest plot of effects on cognitive flexibility[33,34,40,43]**


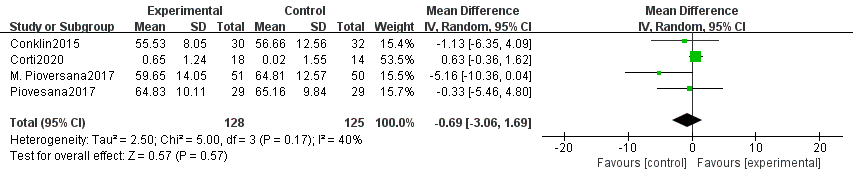


1. **Forest plot of effects on executive function[33,40,43]**


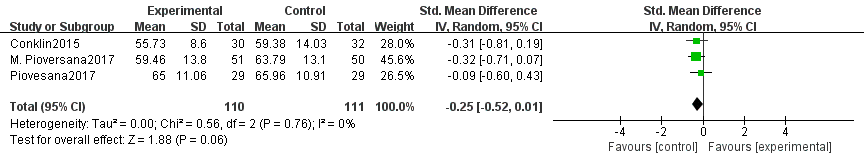

Supplement: Multimedia Appendix 4 [file jmir_v25i1e46957_app4.docx]
