# Supplementary material for: The Effect of Web-Based Telerehabilitation Programs on Children and Adolescents With Brain Injury: Systematic Review and Meta-Analysis
Source: J Med Internet Res. 2023 Dec 25;25:e46957. doi: 10.2196/46957 (PMC10775025; doi:10.2196/46957)

**Multimedia Appendix 5**

**Summary of forest plots for other outcomes**

1. **Forest plot of effects on hand function[32,38,46,47]**


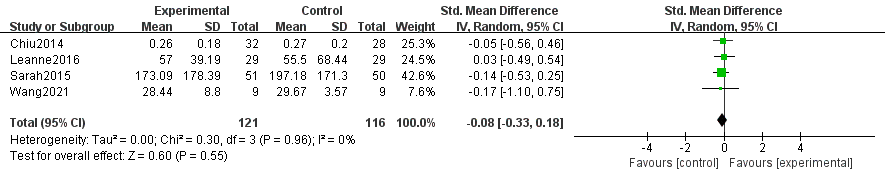


1. **Forest plot of effects on upper limb function[38,46]**


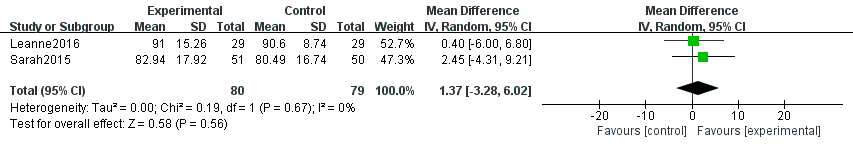


1. **Forest plot of effects on balance function[31,35,39,42]**


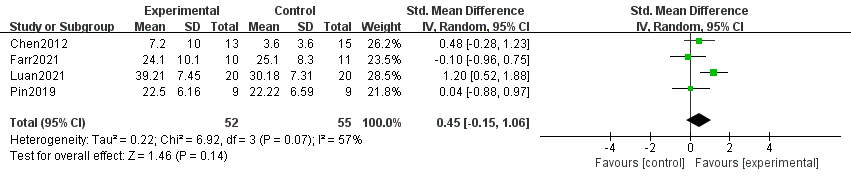


1. **Forest plot of effects on COPM[38,46]**


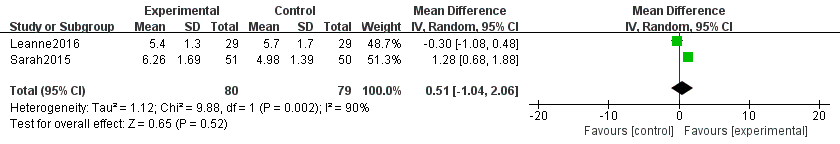

Supplement: Multimedia Appendix 5 [file jmir_v25i1e46957_app5.docx]
