# Supplementary figures and images for: The Effect of Web-Based Telerehabilitation Programs on Children and Adolescents With Brain Injury: Systematic Review and Meta-Analysis
Source: J Med Internet Res. 2023 Dec 25;25:e46957. doi: 10.2196/46957 (PMC10775025; doi:10.2196/46957)

**Multimedia Appendix 6**

**Galbraith plot of the balance outcome[31, 35,39,42]**


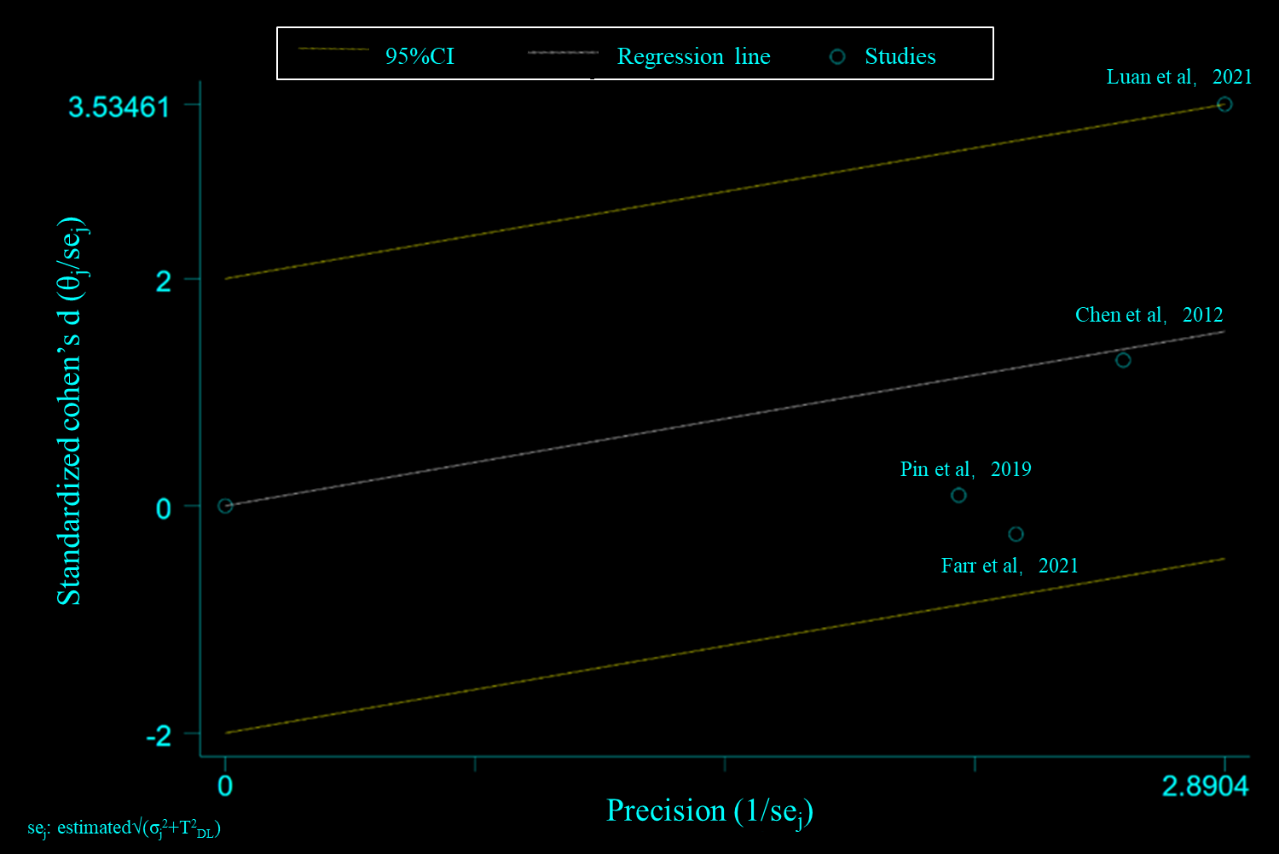

Supplement: Multimedia Appendix 6 [file jmir_v25i1e46957_app6.docx]

**Multimedia Appendix 7**

**Egger's test for motor function**


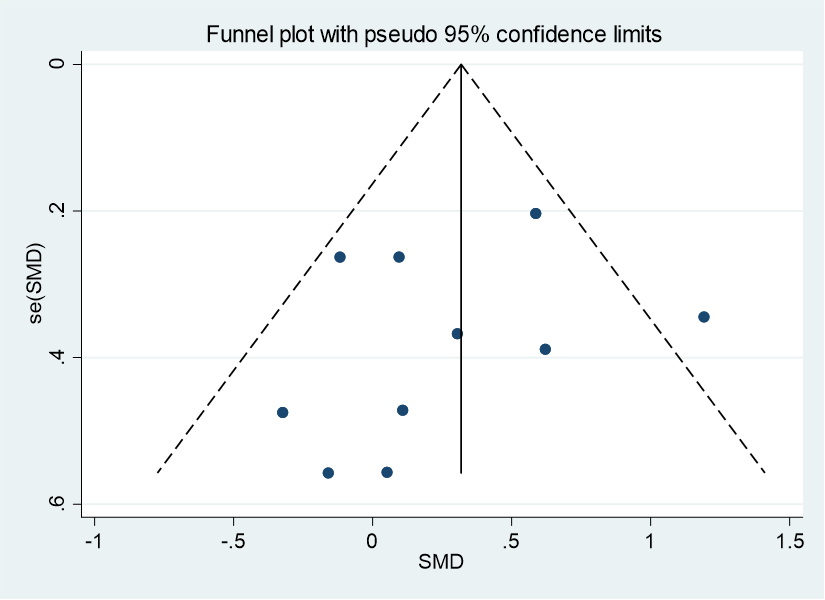


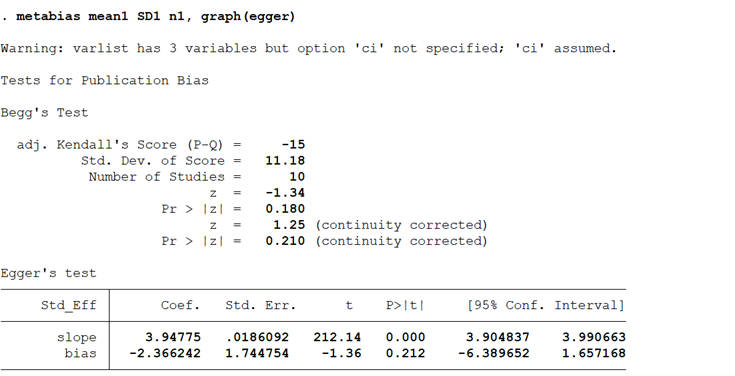

Supplement: Multimedia Appendix 7 [file jmir_v25i1e46957_app7.docx]
