# Supplementary material for: The Effect of Web-Based Telerehabilitation Programs on Children and Adolescents With Brain Injury: Systematic Review and Meta-Analysis
Source: J Med Internet Res. 2023 Dec 25;25:e46957. doi: 10.2196/46957 (PMC10775025; doi:10.2196/46957)
Supplement: Multimedia Appendix 8 [file jmir_v25i1e46957_app8.docx]

**Multimedia Appendix 8**

**GRADE quality evaluation of the outcomes**

| **Certainty assessment** | | | | | | | | **Number of patients** | | **Effect** | | **Certainty** | **Importance** |
| --- | --- | --- | --- | --- | --- | --- | --- | --- | --- | --- | --- | --- | --- |
| **Outcome measures** | **Number of studies** | **Study design** | **Risk of bias** | **Inconsistency** | **Indirectness** | **Imprecision** | **Other considerations** | **Intervention group** | **Waitlist control** | **Relative(95% CI)** | **Absolute(95%CI)** |  |  |
| **Motor function** | **10** | **Randomized trials** | **Very**  **serious** | **Not serious** | **Not serious** | **Not serious** | **strong association** | **189** | **188** | **-** | **SMD 0.29 SD higher**  **(0.01 higher to 0.57 higher)** | **⨁⨁⨁◯**  **Moderate** | **CRITICAL** |
| **Physical activity level** | **5** | **Randomized trials** | **Very**  **serious** | **Not serious** | **Not serious** | **Not serious** | **None** | **115** | **117** | **-** | **SMD 0.42 higher**  **(0.11 higher to 0.73 higher)** | **⨁⨁◯◯ Low** | **CRITICAL** |
| **Lower limb strength** | **3** | **Randomized trials** | **Very**  **serious** | **Not serious** | **Not serious** | **Not serious** | **None** | **89** | **91** | **-** | **SMD 0.52 SD higher**  **(0.13 higher to 0.9 higher)** | **⨁⨁◯◯ Low** | **CRITICAL** |
| **Visual processing skills** | **5** | **Randomized trials** | **Very**  **serious** | **Not serious** | **Not serious** | **Not serious** | **None** | **178** | **172** | **-** | **SMD 0.26 higher**  **(0.02 higher to 0.5 higher** | **⨁⨁◯◯ Low** | **CRITICAL** |
| **Letter-number sequencing** | **2** | **Randomized trials** | **Very**  **serious** | **Not serious** | **Not serious** | **Not serious** | **None** | **81** | **82** | **-** | **SMD 1.26 SD higher**  **(0.26 higher to 2.26 higher)** | **⨁⨁◯◯ Low** | **IMPORTANT** |
| **Arithmetic calculation** | **2** | **Randomized trials** | **Very**  **serious** | **Not serious** | **Not serious** | **Serious** | **None** | **48** | **46** | **-** | **SMD 0.46 SD higher**  **(0.73 lower to 1.65 higher)** | **⨁◯◯◯ Very low** | **IMPORTANT** |
| **Working memory** | **2** | **Randomized trials** | **Very**  **serious** | **Very**  **serious** | **Not serious** | **Serious** | **None** | **48** | **46** | **-** | **SMD 3.59 SD higher**  **(3.1 lower to 10.27 higher)** | **⨁◯◯◯ Very low** | **IMPORTANT** |
| **Attention** | **3** | **Randomized trials** | **Very**  **serious** | **Not serious** | **Not serious** | **Not serious** | **None** | **110** | **111** | **-** | **SMD 0.38 higher**  **(0.09 higher to 0.66 higher)** | **⨁⨁◯◯ Low** | **IMPORTANT** |
| **Coding** | **2** | **Randomized trials** | **Very**  **serious** | **Not serious** | **Not serious** | **Serious** | **None** | **80** | **79** | **-** | **SMD 0.63 SD higher**  **(0.63 lower to 1.9 higher)** | **⨁◯◯◯ Very low** | **IMPORTANT** |
| **Symbol search** | **2** | **Randomized trials** | **Very**  **serious** | **Not serious** | **Not serious** | **Not serious** | **None** | **80** | **79** | **-** | **SMD 1.18 SD higher**  **(0.43 higher to 1.93 higher)** | **⨁⨁◯◯ Low** | **IMPORTANT** |
| **Cognitive flexibility** | **4** | **Randomized trials** | **Very**  **serious** | **Not serious** | **Not serious** | **Serious** | **None** | **128** | **125** | **-** | **SMD 0.1 SD lower**  **(0.4 lower to 0.2 higher)** | **⨁◯◯◯ Very low** | **IMPORTANT** |
| **Executive function** | **3** | **Randomized trials** | **Very**  **serious** | **Not serious** | **Not serious** | **Serious** | **None** | **110** | **111** | **-** | **SMD 0.25 SD lower**  **(0.52 lower to 0.01 higher)** | **⨁◯◯◯ Very low** | **IMPORTANT** |
| **Hand function** | **4** | **Randomized trials** | **Very**  **serious** | **Not serious** | **Not serious** | **Serious** | **None** | **121** | **116** | **-** | **SMD 0.08 SD lower**  **(0.33 lower to 0.18 higher)** | **⨁◯◯◯ Very low** | **IMPORTANT** |
| **Upper limb function** | **2** | **Randomized trials** | **Very**  **serious** | **Not serious** | **Not serious** | **Serious** | **None** | **80** | **79** | **-** | **SMD 1.37 SD higher**  **(3.28 lower to 6.02 higher)** | **⨁◯◯◯ Very low** | **IMPORTANT** |
| **Balance function** | **4** | **Randomized trials** | **Very**  **serious** | **Serious** | **Not serious** | **Serious** | **None** | **52** | **55** | **-** | **SMD 0.45 SD higher**  **(0.15 lower to 1.06 higher)** | **⨁◯◯◯ Very low** | **IMPORTANT** |
| **Occupational performance** | **2** | **Randomized trials** | **Very**  **serious** | **Very**  **serious** | **Not serious** | **Serious** | **None** | **80** | **79** | **-** | **SMD 0.51 SD higher**  **(1.04 lower to 2.06 higher)** | **⨁◯◯◯ Very low** | **IMPORTANT** |
